# Pharmacoepidemiological Research on N-Nitrosodimethylamine-Contaminated Ranitidine Use and Long-Term Cancer Risk: A Population-Based Longitudinal Cohort Study

**DOI:** 10.3390/ijerph191912469

**Published:** 2022-09-30

**Authors:** Chun-Hsiang Wang, I-I Chen, Chung-Hung Chen, Yuan-Tsung Tseng

**Affiliations:** 1 Department of Hepatogastroenterology, Tainan Municipal Hospital (Managed by Show Chwan Medical Care Corporation), Tainan 701033, Taiwan; 2 Department of Optometry, Chung Hwa Medical University, Tainan 701033, Taiwan; 3 Department of Gastroenterology, Chang Bing Show Chwan Memorial Hopital, Changhua 505029, Taiwan; 4 Committee of Medical Research, Tainan Municipal Hospital (Managed by Show Chwan Medical Care Corporation), Tainan 701033, Taiwan

**Keywords:** ranitidine, famotidine, cancers, N-nitrosodimethylamine (NDMA), propensity score matching (PSM)

## Abstract

**Simple Summary:**

There is a lack of published data regarding the association between N-nitrosodimethylamine and human cancer risks. Hence, this study answers several questions using propensity score matching with a large population size selected from a high-quality nationwide and population-based database with a long follow-up period to assess the relationship between the cumulative individual cancer incidence and long-term ranitidine use.

**Abstract:**

N-Nitrosodimethylamine (NDMA), a carcinogenic chemical, has recently been identified in ranitidine. We conducted a population-based study to explore ranitidine use and cancer emergence over time. Using the Taiwan National Health Insurance Research Database, a population-based cohort study was conducted. A total of 55,110 eligible patients who received ranitidine between January 2000 and December 2018 were enrolled in the treated cohort. We conducted a 1:1 propensity-score-matching procedure to match the ranitidine-treated group with the ranitidine-untreated group and famotidine controls for a longitudinal study. The association of ranitidine exposure with cancer outcomes was assessed. A multivariable Cox regression analysis that compared cancer risk with the untreated groups revealed that ranitidine increased the risk of liver (hazard ratio (HR): 1.22, 95% confidence interval (CI): 1.09–1.36, *p* < 0.001), lung (HR: 1.17, CI: 1.05–1.31, *p* = 0.005), gastric (HR: 1.26, CI: 1.05–1.52, *p* = 0.012), and pancreatic cancers (HR 1.35, CI: 1.03–1.77, *p* = 0.030). Our real-world observational study strongly supports the pathogenic role of NDMA contamination, given that long-term ranitidine use is associated with a higher likelihood of liver cancer development in ranitidine users compared with the control groups of non-ranitidine users treated with famotidine or proton-pump inhibitors.

## 1. Introduction

Ranitidine, a histamine-2 receptor antagonist, inhibits gastric acid secretion when treating gastroesophageal reflux disease and peptic ulcers [1]. Additionally, according to the data from the Food and Drug Administration (FDA) Adverse Event Reporting System, elevated and significant proportional reporting ratios (PRRs) were observed for pharyngeal, esophageal, stomach, colorectal, liver, and pancreatic cancers, including elevated PRRs for anal and gallbladder cancers [2].

In 2019, the U.S. FDA declared that N-nitrosodimethylamine (NDMA), with the formula (CH3)2NNO, identified in medicines containing valsartan and ranitidine, is a member of N-nitrosamines and a known carcinogen, according to laboratory results [3,4,5]. The FDA’s testing of ranitidine products revealed that NDMA levels were nine times greater than the FDA’s recommended limit, resulting in global recalls [6,7]. Several studies have also reported that NDMA could be oncogenic in animals [8]. According to the International Agency for Research on Cancer report, NDMA has been proven to belong to group 2A and to be “probably carcinogenic to humans” [9].

A study also reported that high ranitidine doses combined with nitrite produced DNA fragmentation in rodents’ livers and gastric mucosa [10]. Many observational human studies have reported that consuming a high number of NDMA-contaminated foods may be linked to an increased risk of stomach and colon cancers [11,12]. Additionally, detailed experimental animal studies showed that cancer risk may increase with NDMA exposure through inhalation or oral delivery and that tumors developed in the lungs, liver, kidneys, and bile ducts in animals [13]. Several studies previously examined the carcinogenic effects of NDMA on humans, although they were equivocal. Some authors reported no association between ranitidine use and cancer risk [14,15,16], whereas others supported the connection [17,18,19,20]. In addition to cancers caused by NDMA contamination, multiple studies reported that acid-suppressive agents, such as proton-pump inhibitors (PPIs) and histamine-2 receptor antagonists (H2RAs), were linked to gastric [21,22] and liver cancers [23,24,25,26]. However, these reports were contradictory, and the data were not sufficient to reach definite conclusions. The conflicting results of studies underlie the lack of concrete evidence supporting the role of ranitidine in cancer development. Therefore, we aimed to conduct a large-scale, long-term follow-up cohort study to investigate ranitidine use and the subsequent emergence of cancer over time in a real-life setting.

## 2. Materials and Methods

### 2.1. Data Source

This study used Taiwan’s National Health Insurance Database (NHIRD), which is a population-based claims database, and a cross-sectional survey participated in by over 99% of Taiwan’s population. We included all medical services, procedures, and prescription medication data from 1 January 2000 to 31 December 2018. The diagnoses recorded in the NHIRD are coded in accordance with the International Classification of Disease, Ninth Revision, Clinical Modification (ICD-9-CM), and Tenth Revision, Clinical Modification (ICD-10-CM).

Considering that the NHIRD dataset consists of encrypted secondary data, each person is impossible to identify; thus, the informed consent requirement was waived. The Research Ethics Committee of Show Chwan Memorial Hospital approved the study protocol on 14 December 2021 (IRB-No: 1101105).

### 2.2. Study Design and Study Participants

The total doses for each ranitidine prescription during the follow-up period were calculated to indicate the duration of ranitidine exposure. As the World Health Organization proposed, one defined daily dose (DDD) of ranitidine was 300 mg/day [27]. We defined 90 DDDs as the valid treatment for patients with reflux esophagitis and peptic ulcer disease treated with 300 mg ranitidine daily for 3 months [28,29]. Patients prescribed ranitidine at ≥90 DDDs were assigned to the ranitidine cohort, whereas those who never used ranitidine belonged to the non-ranitidine cohort.

We investigated whether a dose–response relationship exists between ranitidine and cancer diagnosis. For the sensitivity analysis, we grouped the patient follow-up period into four intervals according to the cumulative dose, starting from the first prescription: 90–180, 181–270, 271–360, and >360 DDDs.

### 2.3. Potential Confounders

The exclusion criteria were as follows: age < 40 years, diagnosis with cancer before the index date, ranitidine use <90 DDDs, and follow-up <1 year.

We enrolled ranitidine users who were matched for exact age, sex, the Charlson comorbidity index (CCI), comorbidities (hypertensive cardiovascular disease (HCD0, hyperlipidemia, diabetes mellitus (DM), and chronic kidney disease (CKD)), medications (aspirin, statins, angiotensin-converting enzyme inhibitors (ACEIs), β-blockers, spironolactone, glucocorticoids, and selective serotonin reuptake inhibitors (SSRIs), and antiviral therapy for hepatitis B or C virus (HBV and HCV, respectively) infection), and the index date.

The final matched cohort consisted of 55,110 patients who were evaluated from the index date until the target cancer onset, death, or the end of the study period (31 December 2018). 

### 2.4. Covariate Assessment

The main confounding factors were based on a recent study investigating the association between N-nitrosodimethylamine and cancer. We adjusted for the following covariates that potentially affect cancer incidence: age, sex, and medication history (low-dose aspirin, statins, ACEIs, β-blockers, spironolactone, glucocorticoids, SSRIs, and antiviral treatment for chronic hepatitis B or C). 

We also considered the following comorbidities: HCD (ICD-9 codes 401–405; ICD-10 codes I10–I15), hyperlipidemia (ICD-9 code 272; ICD-10 code E78), DM (ICD-9 code 250; ICD-10 codes E10.0, E10.1, E10.9, E11.0, E11.1, and E11.9), CKD (ICD-9 code 585; ICD-10 code N18), and CCI.

### 2.5. Main Outcome Measurements

We assessed the following cancer categories for the first cancer diagnosis: liver cancer (ICD-9 code 155; ICD-10 code C22), oral cancer (ICD-9 codes 140–149; ICD-10 codes C00–C14), esophageal cancer (ICD-9 code 150; ICD-10 code C15), gastric cancer (ICD-9 code 151; ICD-10 code C16), colon cancer (ICD-9 code 153; ICD-10 code C18), rectal cancer (ICD-9 code 154; ICD-10 codes C19–C21), pancreatic cancer (ICD-9 code 157; ICD-10 code C25), lung cancer (ICD-9 code 162; ICD-10 codes C33 and C34), bone cancer (ICD-9 code 170; ICD-10 codes C40 and C41), bladder cancer (ICD-9 code 188; ICD-10 code C67), renal cancer (ICD-9 code 189; ICD-10 codes C65, C66, and C68), thyroid cancer (ICD-9 code 193; ICD-10 code C73), skin cancer (ICD-9 codes 172–173; ICD-10 code C44), breast cancer in females (ICD-9 code 174; ICD-10 code C50), uterine cancer (ICD-9 code 179; ICD-10 code C55), cervical cancer (ICD-9 code 180; ICD-10 code C53), ovarian cancer (ICD-9 code 183; ICD-10 codes C56 and C570–C574), prostate cancer (ICD-9 code 185; ICD-10 codes C61), and overall cancer. For breast, uterine, cervical, and ovarian cancers, the analysis was restricted to females, and prostate cancer was restricted to males.

### 2.6. Exposure Definition and Follow-Up

In this study, we adopted a new-user design with 1 year as the washout period to eliminate the influence of external factors in patients with newly diagnosed cancer [30]. Patients with no predefined outcomes or those who died during the follow-up were censored. We defined the index date for each ranitidine user as the date of their first prescription. For their corresponding matched comparison group, the index date was set to be that of their matched individual with ranitidine use. All patients were followed up from the index date to 2018. The mean follow-up period was 9.56 ± 5.96 (median: 8.42) years for the ranitidine cohort and 9.70 ± 5.96 (median: 8.58) years for the non-ranitidine cohort. All prescriptions, diagnostic outcomes, and deaths were ascertained until 31 December 2018. For all groups, the follow-up duration was defined as the interval from the date of enrollment to the date of cancer diagnosis, death, or the end of the follow-up period, whichever came first.

### 2.7. Statistical Analysis

Categorical variables were compared using the McNemar test. Continuous variables such as the prescription and medical records as baseline characteristics were compared using paired *t*-tests. To reduce potential selection bias, we used propensity score matching (PSM) to balance the differences in proportions, such as comorbidities, between the ranitidine and non-ranitidine cohorts.

For a robust propensity score matching, 1:1 full matching without replacement was performed. Therefore, the regression model was specified correctly relative to the population regression function of the outcome variable on the treatment and all covariates used for matching. Therefore, trimming techniques were not employed in the weighting approach [31,32]. PSM was performed using multivariate logistic regression analysis and nearest-neighbor matching with the R package “MatchIt” (version 4.3.4).

We used Cox proportional hazards regression models to estimate the hazard ratios (HRs) and 95% confidence intervals (CIs) of cancer risk for ranitidine users in comparison with non-ranitidine users. To confirm the stability and robustness of our model, all hazard ratios and their 95% CIs were modified using the bootstrapping method [33,34]. The outcomes of the different study cohorts were estimated using the Kaplan–Meier method, and the differences in curves were examined using the log-rank test. Sensitivity analysis was conducted to validate the individual events from the index date to the end of the study in different DDD exposure groups using Cox proportional hazards regression models and the Kaplan–Meier method.

The competing risks of death were adjusted using the R package “cmprsk” (version 2.2–11), and the regression model was assessed according to Fine and Gray. All data management procedures were performed using SPSS 21.0 (SPSS Inc., Chicago, IL, USA) and R version 3.4.3 (R Core Team, 2017). A *p*-value of <0.05 was considered statistically significant.

## 3. Results

**Figure 1:**
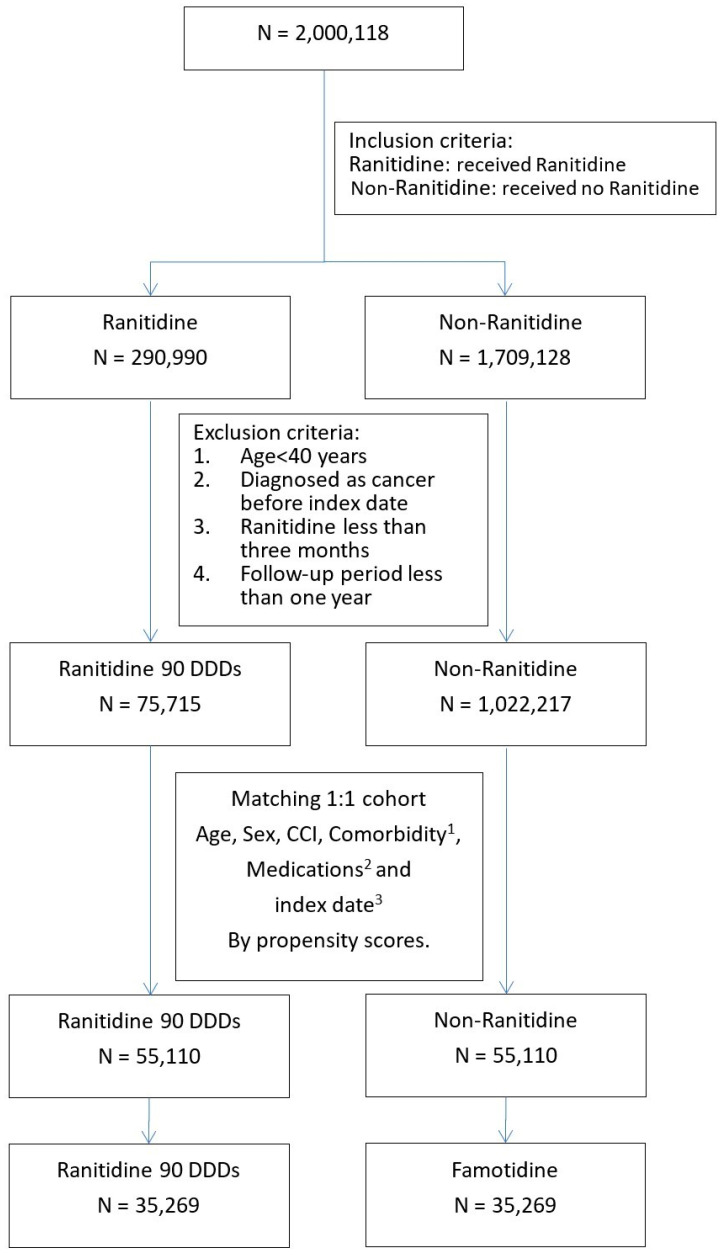
Flowchart of the selection of study patients. ^1^ Comorbidities: hypertensive cardiovascular disease, hyperlipidemia, diabetes mellitus, and chronic kidney disease. ^2^ Medications: aspirin, statins, angiotensin-converting enzyme inhibitors, β-blockers, famotidine, spironolactone, glucocorticoids, selective serotonin reuptake inhibitors, and antiviral therapy for hepatitis B or C. ^3^ Index date: exact date of the first prescription.

In the NHIRD database, we found 290,990 ranitidine users and 1,709,128 non-ranitidine users within the study period (Figure 1). In accordance with the exclusion criteria, 75,715 and 1,022,217 patients were finally included in the ranitidine and non-ranitidine cohorts, respectively. Using PSM, we matched the ranitidine cohort (*n* = 55,110) with the non-ranitidine cohort (*n* = 55,110) in a 1:1 model. Figure 1 illustrates the flowchart of patient selection.

Patients from the non-ranitidine cohort who were statistically matched with those from the ranitidine cohort were selected in consideration of the following factors: age, sex, CCI, and comorbidities, including HCD, hyperlipidemia, DM, and CKD.

Certain medications (aspirin, statins, ACEIs, β-blockers, spironolactone, glucocorticoids, SSRIs, and antiviral therapy for HBV or HCV infection) and the index date (the exact date of diagnosis) were potential confounders for most cancers. Table 1 shows the baseline characteristics of the well-balanced, 1:1 matched cohort. Sex, age, CCI scores, and the follow-up period were fully matched between the ranitidine and non-ranitidine cohorts. Overall, the male–female ratio was 47.8:52.2 (*p* = 1.000), with mean values of 66.8 ± 14.1 (*p* = 1.000), 3.77 ± 2.78 (*p* = 1.000), and 9.2 ± 5.9 years (*p* = 1.000) for age, CCI, and follow-up duration, respectively. The median follow-up duration was 8.3 years.

**Figure 2:**
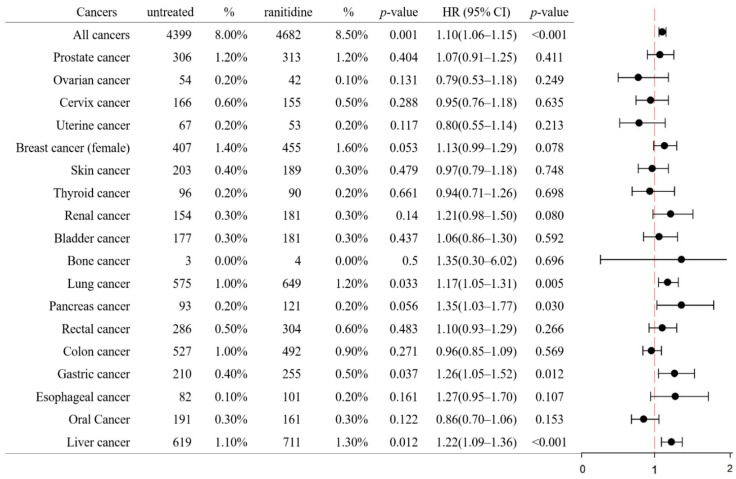
Number and proportion of 18 different cancer reports in the ranitidine and non-ranitidine cohorts and the associated proportional reporting ratios and 95% confidence intervals in multivariate Cox proportional hazards regression adjusted for competing mortality.

**Figure 3:**
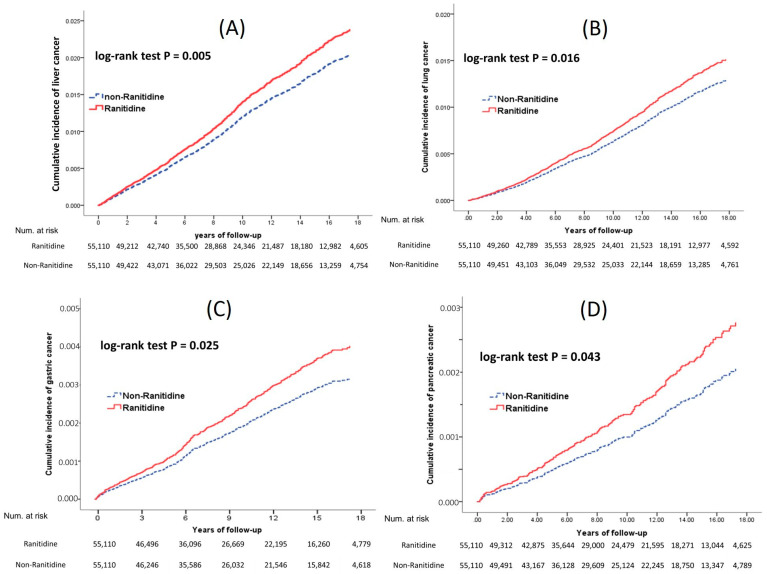
Cumulative incidences of single cancers after adjustment for competing risks. (**A**) Liver cancer, (**B**) lung cancer, (**C**) gastric cancer, and (**D**) pancreatic cancer.

As shown in Figure 2, the ranitidine cohort showed a significantly higher prevalence of liver cancer (1.1% vs. 1.3%; *p* = 0.012), gastric cancer (0.4% vs. 0.5%; *p* = 0.037), and lung cancer (1.0% vs. 1.2%; *p* = 0.033) and a higher overall cancer rate (8.0% vs. 8.5%; *p* = 0.001) than the non-ranitidine cohort. In terms of the cumulative incidence rates and HRs, ranitidine use was associated with overall cancer (HR, 1.10; 95% CI, 1.06–1.15), liver cancer (HR, 1.22; 95% CI, 1.09–1.36), gastric cancer (HR, 1.26; 95% CI, 1.05–1.52), pancreatic cancer (HR, 1.35; 95% CI, 1.03–1.77), and lung cancer (HR, 1.17; 95% CI, 1.05–1.31) compared with non-ranitidine use. No significant associations were observed for the 14 other cancers. In the Kaplan–Meier analysis, the ranitidine cohort exhibited a significantly higher risk of developing liver cancer (log-rank test, *p* = 0.005), lung cancer (*p* = 0.016), gastric cancer (*p* = 0.025), and pancreatic cancer (*p* = 0.043) than the non-ranitidine cohort (Figure 3).

Table 2 shows the incidence of each cancer. The incidence rate was 9.19 per 1000 person-years in the ranitidine group and 8.49 per 1000 person-years in the non-ranitidine group. The incidence rates of certain cancers were greater in the group exposed to ranitidine than in the unexposed group. Ranitidine use was associated with some individual cancers with a high incidence rate (per 1000 person-years); these cancers were liver (1.35 vs. 1.16), lung (1.23 vs. 1.07), gastric (0.48 vs. 0.39), and pancreatic (0.23 vs. 0.17) cancers.

### 3.1. Ranitidine Duration Effect on Cancer Development

The effect of ranitidine use on the risk of progression to significant individual cancers (e.g., liver cancer, gastric cancer, lung cancer, and pancreatic cancer) was assessed by multivariate Cox regression analysis adjusted for age, sex, CCI, co-medications (aspirin, statins, ACEIs, β-blockers, spironolactone, glucocorticoids, SSRIs, and antiviral therapy for HBV or HCV infection), comorbidities (HCD, hyperlipidemia, DM, and CKD), and the calendar date at the start of follow-up. 

**Figure 4:**
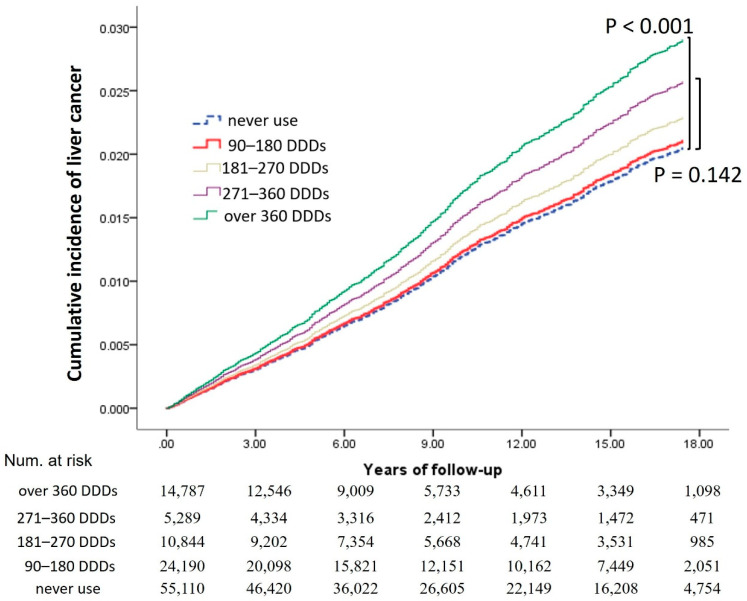
Cumulative incidences of liver cancer for different prescription durations after adjustment for competing risks. Abbreviations: DDDs, defined daily doses.

Ranitidine users were divided into groups according to drug exposure: 90–180 DDDs, 181–270 DDDs, 271–360 DDDs, >360 DDDs, and an unexposed group. After adjusting for potential confounders, we found that ranitidine use was a potential risk factor for liver cancer development. For patients with relatively limited exposure to ranitidine (<360 DDDs), ranitidine did not significantly affect the risk of developing liver cancer compared with nonusers (Figure 4). However, increased ranitidine exposure was associated with liver cancer risk. For patients with >360 DDDs, the adjusted HR of liver cancer development was 1.42 (95% CI: 1.22–1.66; *p* < 0.001) compared with that in the non-ranitidine group (Table 3). 

Regression was adjusted for age, sex, the Charlson comorbidity index, co-medications (aspirin, statins, angiotensin-converting enzyme inhibitors, β-blockers, spironolactone, glucocorticoids, selective serotonin reuptake inhibitors, and antiviral therapy for hepatitis B or C), comorbidities (hypertensive cardiovascular disease, hyperlipidemia, diabetes mellitus, and chronic kidney disease), and calendar year at the start of follow-up.

### 3.2. Comparison between Ranitidine and Famotidine for the Association with Patient Outcomes

To avoid potential indication bias, we selected non-ranitidine users (control subjects) by PSM for the famotidine cohort, with a ranitidine–famotidine ratio of 1:1. This subgroup was added to determine whether the use of ranitidine increases the risk of developing cancers due to the related indication.

We screened the risk for cancer in the ranitidine (*n* = 35,269) and famotidine (*n* = 35,269) cohorts (Figure 1). Ranitidine users who were statistically matched with famotidine users were selected, adjusting for the following factors: age, sex, indications, co-medications, and comorbidities. 

The prevalence of overall cancer was 3052 (8.7%) in the ranitidine group and 2924 (8.3%) in the famotidine group. The overall cancer risk was statistically different between these two groups (adjusted HR, 1.07; 95% CI, 1.02–1.12, *p* = 0.010). Significant differences were also observed in liver (adjusted HR, 1.22; 95% CI, 1.06–1.40, *p* = 0.005) and renal cancer (adjusted HR, 1.33; 95% CI, 1.02–1.73, *p* = 0.034) outcomes between the two groups (Table 4).

**Figure 5:**
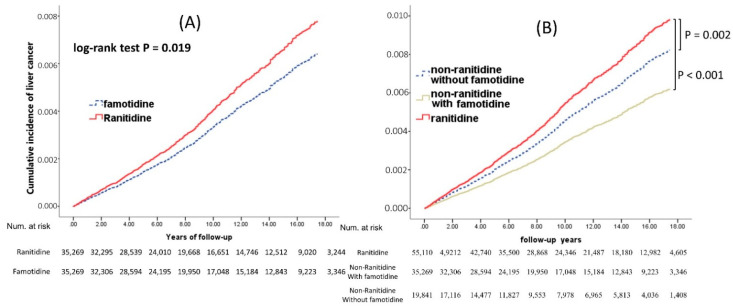
(**A**) Cumulative incidences of liver cancer between ranitidine and famotidine users after adjustment for competing risks. (**B**) Cumulative incidences of liver cancer among ranitidine, non-ranitidine with famotidine, and non-ranitidine without famotidine users after adjustment for competing risks.

In the Kaplan–Meier analysis, we found that liver cancer risk was significantly different between the ranitidine and famotidine cohorts with a balanced model (*p* = 0.019, Figure 5A). Moreover, the liver cancer risk was significantly higher in the ranitidine cohort than in non-ranitidine with famotidine and non-ranitidine without famotidine cohorts (*p* < 0.001 and *p* = 0.02, respectively; Figure 5B). Therefore, the pattern of the cumulative incidence of liver cancer was the same in the non-ranitidine groups, regardless of famotidine use.

### 3.3. Comparison between Ranitidine and PPIs for Their Association with Liver Cancer

Considering another potential indication bias, we categorized the non-ranitidine users (control subjects) into those with and without PPI use. This subgroup analysis aimed to determine whether ranitidine use increased liver cancer risk due to an alternative medicine with a related indication.

**Figure 6:**
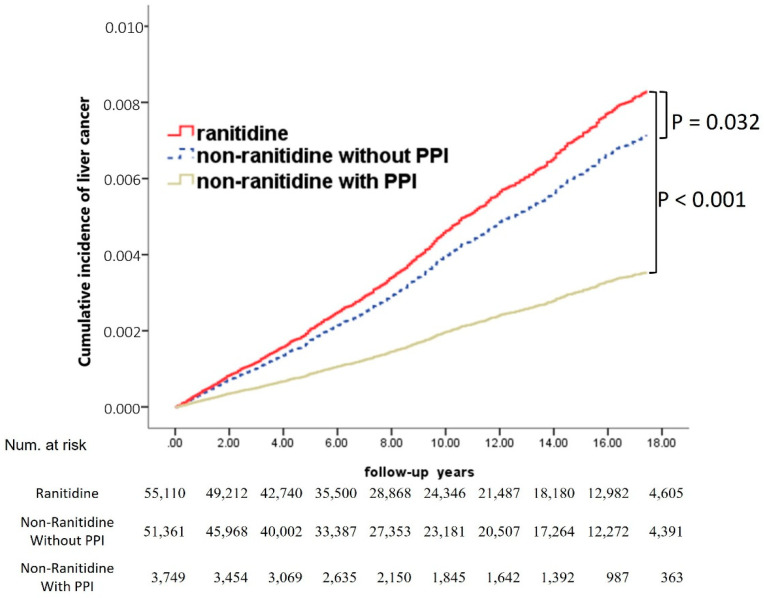
Cumulative incidences of liver cancer among ranitidine, non-ranitidine with PPI, and non-ranitidine without PPI users after adjustment for competing risks.

We screened the risk for cancer in the ranitidine (*n* = 55,110), ranitidine without PPI (*n* = 51,361), and ranitidine with PPI (*n* = 3749) cohorts (Figure 6). Ranitidine users were selected to adjust for the following factors: age, sex, indications, co-medications, and comorbidities. 

The liver cancer risk was significantly higher in the ranitidine group than in the non-ranitidine without PPI group (adjusted HR, 1.16; 95% CI, 1.04–1.30, *p* = 0.006). Furthermore, liver cancer risk was significantly lower in the non-ranitidine with PPI group than in the non-ranitidine without PPI group (adjusted HR, 0.49; 95% CI, 0.33–0.75, *p* = 0.001). In the Kaplan–Meier analysis, we found that liver cancer risk was significantly higher in the ranitidine group than in the non-ranitidine without PPI and non-ranitidine with PPI groups (*p* = 0.032 and *p* < 0.001, respectively; Figure 6).

## 4. Discussion

The current research, a population-level epidemiologic study, evaluated cancer risk attributed to long-term ranitidine use with NDMA exposure, which was linked to a higher liver cancer risk than the non-ranitidine group and the famotidine group. Several epidemiological analyses have reported the public health concern of NDMA exposure, which has been linked to an increased risk of stomach and colon cancers [11,12,35]. The carcinogenic effects of NDMA theoretically result from inducing DNA-damaging metabolites in the gastrointestinal tract and liver, as suggested by animal studies. Notably, NDMA is metabolized in the liver by CYP2E1 to methyl diazonium, leading to mutations caused by methylation and the development of liver cancer [3,36]. Several studies have reported that hypoacidity due to acid-suppressive medication use also plays a critical role in the development of liver and gastric cancers. The hypothesized mechanisms include bacterial overgrowth, the formation of N-nitroso compounds, lipopolysaccharides, and deoxycholic acid, which have been linked to the development of liver cancer [37,38,39,40,41,42,43]. Additionally, higher gastrin levels following PPI or H2RA use may be associated with gastrointestinal malignancies [44,45]. Therefore, it is reasonable to assume that a link between acid-suppressive medication use and cancer development may be based on differing mechanisms. However, the clear data from our real-world observational study strongly support the pathogenic role of NDMA contamination, given that long-term ranitidine use is associated with a higher likelihood of cancer development in ranitidine users compared to the control groups of non-ranitidine users who were treated with PPIs or famotidine. Conversely, an increasing number of recent clinical and epidemiological studies [14,15] concluded that there is no convincing evidence of the carcinogenic potency of ranitidine. Nevertheless, the limitations of the two studies mentioned above should be considered since their small sample size and short follow-up duration may cause statistical bias and inaccurate conclusions. One notable strength of our study is the huge population size selected from a high-quality nationwide and population-based database with a long follow-up period of 18 years. Specifically, it was based on a cohort design of a seemingly prospective technique to explore ranitidine exposure and cancer outcomes. Additionally, outcome data were retrieved from formal cancer registries, which are more accurate than other sources. Using PSM, our study constructed an artificial control group (non-ranitidine users) with similar characteristics by combining it with additional matching for multiple prognostic factors or regression adjustment. Using these matches, we estimated the impact of ranitidine intervention on cancer risk, which showed increased odds of developing liver, lung, pancreatic, and gastric cancers. The Kaplan–Meier analysis of our 18-year dataset confirmed these findings.

We included a second active comparator group of individuals who were also prescribed famotidine, containing no NDMA and used for an almost identical indication, which might minimize potential bias to clarify potential confounding by indication.

The overall cancer risk was statistically different between these two groups compared with famotidine or non-ranitidine users. Notably, liver and renal cancers were more common among ranitidine users. Furthermore, the Kaplan–Meier analysis revealed that liver cancer risk was significantly higher in the ranitidine cohort than in the famotidine cohort. Our study observed this outcome using non-ranitidine users as a control group. Additionally, this result contradicts other reports [14,15]. Therefore, based on a direct comparison with either the non-ranitidine group or the famotidine group (similar indication to ranitidine users), only liver cancer displayed a significant association with long-term ranitidine use. This approach was used to ameliorate the implicit indication bias that occurs when the cancer risk is related to the indication for medication use but not to the use of the medication itself [46]. 

Another comparative approach in our study revealed the association of ranitidine usage with four individual cancers with a high incidence rate (per 1000 person-years), including liver, lung, gastric, and pancreatic cancers, compared with the general population. However, as stated by Roberts et al. [27,47,48], the preference for a pharmacoepidemiological study of drug safety is to develop a new-user design rather than a prevalent-user design in which patients have already been receiving therapy for some time before the study follow-up begins. Therefore, our study used 1 year as the washout period, during which the participants were taken off ranitidine, to remove the effects of treatment before the study initiation. Nonetheless, ranitidine still showed similar results after excluding “protopathic bias” [49], meaning that ranitidine use sometimes precedes cancer development before it is diagnosed.

A positive quality of our study is the use of PSM [50] in a population-based cohort design, which imitates a randomized trial to control for confounding factors that mostly depend on selecting the documented confounders used in the matching model [51]. These potential confounders in our study, which were causally associated with cancer development, include age, sex, CCI, comorbidities, and medications. Nonetheless, PSM cannot specifically balance unknown factors as randomized controlled trials (RCTs) do. Therefore, some experts [52] argued that substantial bias exists in a PSM study, which is one of our study limitations. However, despite having this limitation, PSM potentially takes advantage of the ability to generate a huge sample size from a large database within a short time. Moreover, it is impractical and unethical to conduct an RCT to test the carcinogenicity of ranitidine in a patient.

In an additional dose–response subanalysis, given that drug–cancer associations are mostly dose-dependent, we further stratified the extent of NDMA exposure by cumulative ranitidine usage based on drug exposure: 90–180 DDDs, 181–270 DDDs, 271–360 DDDs, >360 DDDs, and an unexposed group. Notably, when considering the dose–response of ranitidine usage, there were significant trends of increased liver cancer risk with an increasing dose of ranitidine. However, there was no continuous dose–response relationship among the other individual cancers. Additionally, Iwagami et al. [14] reported contrary results, although they acknowledged a weakness in the study design due to the limited sample size and statistical power. 

The conclusive results of our study after gathering data emphasize that consuming high levels of NDMA due to ranitidine use is linked to liver cancer development. Many current pieces of evidence based on several animal studies propose that NDMA affects liver cancer development, mostly originating from a detailed exploration of the molecular basis of NDMA’s carcinogenic action [53,54,55,56,57]. For example, Souliotis et al. [58] reported that rats exposed to hepatocarcinogenic NDMA (0.2–2.64 ppm in the drinking water) for up to 180 days had a rapid accumulation of N7- and O6-methylguanine in the liver and white blood cells. The analysis of DNA, with the maximum adduct levels reached within 1–7 days dose-dependently, indicates that the accumulation of DNA damage and alterations in hepatocyte DNA replication during chronic NDMA exposure may influence the dose dependence of its carcinogenic efficacy. Notably, in the actual scenario, our result agrees with the above experimental data on the cumulative dose of ranitidine usage, which plays a vital role in hepatocarcinogenesis.

The present study has several limitations. First, it was constrained by the study design since we could not accurately estimate the NDMA level. Second, there were no data available in our database regarding certain confounders, such as alcohol consumption and cigarette smoking. Third, the patients’ medication compliance cannot be detected from the NHIRD. Fourth, there was scarce information regarding over-the-counter ranitidine usage, which caused the underestimation of ranitidine exposure. Fifth, the NHIRD data used in our study was not the most recent. Sixth, the NHIRD lacks specific laboratory information. Finally, potential misdiagnosis, including comorbidities and cancer categories, is possible in the NHIRD due to the potential misclassification of ICD-9-CM and ICD-10-CM codes.

## 5. Conclusions

To conclude, the clinically meaningful results of this large-scale, longitudinal population-based cohort study using an excellent prescription and cancer database provide concrete evidence with very convincing long-term follow-up information for exploring the causative role of ranitidine in increasing the risk of carcinogenic effects on the liver, which was primarily caused by increasingly heavier ranitidine usage. However, to elucidate the underlying mechanisms of its causal association, further studies are necessary.

## Figures and Tables

**Table 1:** Baseline characteristics of the non-ranitidine cohort and ranitidine cohort with over 90 DDDs.

Characteristics		Untreated *n* = 55,110	%	Ranitidine *n* = 55,110	%	*p*-Value
Sex	Female	28,794	52.2%	28,794	52.2%	1.000
	male	26,316	47.8%	26,316	47.8%	
Age (mean ± SD)		66.8 ± 14.1		66.8 ± 14.1		1.000
CCI	0–1	12,444	22.6%	12,444	22.6%	1.000
	2–3	16,205	29.4%	16,205	29.4%	
	4–5	12,799	23.2%	12,799	23.2%	
	>5	13,662	24.8%	13,662	24.8%	
HCD	No	22,204	40.3%	22,204	40.3%	1.000
	Yes	32,906	59.7%	32,906	59.7%	
Hyperlipidemia	No	26,072	47.3%	26,072	47.3%	1.000
	Yes	29,038	52.7%	29,038	52.7%	
DM	No	37,598	68.2%	37,598	68.2%	1.000
	Yes	17,512	31.8%	17,512	31.8%	
CKD	No	49,704	90.2%	49,704	90.2%	1.000
	Yes	5406	9.8%	5406	9.8%	
Aspirin	No	25,810	46.8%	25,810	46.8%	1.000
	Yes	29,300	53.2%	29,300	53.2%	
Statins	No	32,089	58.2%	32,089	58.2%	1.000
	Yes	23,021	41.8%	23,021	41.8%	
ACEIs	No	31,214	56.6%	31,214	56.6%	1.000
	Yes	23,896	43.4%	23,896	43.4%	
β-Blockers	No	16,047	29.1%	16,047	29.1%	1.000
	Yes	39,063	70.9%	39,063	70.9%	
Famotidine	No	19,841	36.0%	19,841	36.0%	1.000
	Yes	35,269	64.0%	35,269	64.0%	
Spironolactone	No	48,320	87.7%	48,320	87.7%	1.000
	Yes	6790	12.3%	6790	12.3%	
Glucocorticoids	No	5748	10.4%	5748	10.4%	1.000
	Yes	49,362	89.6%	49,362	89.6%	
SSRIs	No	44,749	81.2%	44,749	81.2%	1.000
	Yes	10,361	18.8%	10,361	18.8%	
Antiviral therapy	No	54,271	98.5%	54,271	98.5%	1.000
	Yes	839	1.5%	839	1.5%	

Abbreviations: DDDs, defined daily doses; SD, standard deviation; CCI, Charlson comorbidity index; HCD, hypertensive cardiovascular disease; DM, diabetes mellitus; CKD, chronic kidney disease; ACEI, angiotensin-converting enzyme inhibitor; SSRIs, selective serotonin reuptake inhibitors.

**Table 2:** Incidence rates of individual cancers (per 1000 person-years).

Cancers	Untreated	Incidence Rate *	(95% CI)	Ranitidine	Incidence Rate *	(95% CI)
Liver cancer	619	1.16	(1.07–1.25)	711	1.35	(1.25–1.45)
Oral Cancer	191	0.36	(0.30–0.41)	161	0.30	(0.26–0.36)
Esophageal cancer	82	0.15	(0.12–0.19)	101	0.19	(0.16–0.23)
Gastric cancer	210	0.39	(0.34–0.44)	255	0.48	(0.43–0.55)
Colon cancer	527	0.99	(0.90–1.07)	492	0.93	(0.85–1.02)
Rectal cancer	286	0.53	(0.47–0.60)	304	0.58	(0.51–0.64)
Pancreas cancer	93	0.17	(0.14–0.21)	121	0.23	(0.19–0.27)
Lung cancer	575	1.07	(0.98–1.16)	649	1.23	(1.14–1.33)
Bone cancer	3	0.01	(0.00–0.02)	4	0.01	(0.00–0.02)
Bladder cancer	177	0.33	(0.28–0.38)	181	0.34	(0.30–0.40)
Renal cancer	154	0.29	(0.25–0.33)	181	0.34	(0.30–0.40)
Thyroid cancer	96	0.18	(0.14–0.21)	90	0.17	(0.14–0.21)
Skin cancer	203	0.38	(0.33–0.43)	189	0.36	(0.31–0.41)
Breast cancer (female)	407	0.76	(0.69–0.83)	455	0.86	(0.79–0.95)
Uterine cancer	67	0.12	(0.09–0.15)	53	0.10	(0.08–0.13)
Cervix cancer	166	0.31	(0.27–0.36)	155	0.29	(0.25–0.34)
Ovarian cancer	54	0.10	(0.08–0.13)	42	0.08	(0.06–0.11)
Prostate cancer	306	0.57	(0.51–0.63)	313	0.59	(0.53–0.66)
All cancers	4399	8.49	(8.24–8.73)	4682	9.19	(8.93–9.45)

* Incidence rate (per 1000 person-years).

**Table 3:** Estimates for the association between ranitidine use duration and cancer risk compared with non-ranitidine use by multivariate Cox proportional hazards regression.

	Liver Cancer	*p*	Gastric Cancer	*p*	Lung Cancer	*p*	Pancreatic Cancer	*p*
Never used	1.00		1.00		1.00		1.00	
90–180 DDDs *	1.03 (0.89–1.18)	0.690	1.26 (1.00–1.59)	0.049	1.25 (1.09–1.44)	0.002	1.64 (1.19–2.26)	0.003
181–270 DDDs	1.12 (0.93–1.34)	0.220	1.13 (0.82–1.54)	0.452	1.09 (0.90–1.32)	0.403	1.10 (0.69–1.77)	0.682
271–360 DDDs	1.26 (0.99–1.61)	0.064	1.27 (0.84–1.93)	0.252	1.31 (1.02–1.68)	0.032	0.92 (0.45–1.89)	0.816
Over 360 DDDs	1.42 (1.22–1.66)	<0.001	1.33 (1.02–1.74)	0.037	1.04 (0.87–1.24)	0.658	1.22 (0.80–1.85)	0.358

* Abbreviations: DDDs, defined daily doses.

**Table 4:** Risk of cancer between famotidine and ranitidine users.

Cancers	Famotidine	%	Ranitidine	%	Total	*p*-Value	HR (95% CI)	*p*-Value
Liver cancer	380	1.1%	442	1.3%	822	0.032	1.22(1.06–1.40)	0.005
Oral cancer	125	0.4%	107	0.3%	232	0.237	0.87(0.67–1.12)	0.286
Esophageal cancer	52	0.1%	60	0.2%	112	0.451	1.19(0.82–1.72)	0.364
Gastric cancer	142	0.4%	165	0.5%	307	0.208	1.19(0.95–1.49)	0.122
Colon cancer	365	1.0%	309	0.9%	674	0.033	0.86(0.74–1.01)	0.059
Rectal cancer	193	0.5%	195	0.6%	388	0.919	1.03(0.84–1.26)	0.768
Pancreas cancer	66	0.2%	80	0.2%	146	0.281	1.25(0.90–1.73)	0.186
Lung cancer	356	1.0%	400	1.1%	756	0.116	1.15(1.00–1.33)	0.052
Bone cancer *	n/a	n/a	n/a	n/a	n/a	n/a	1.51(0.25–9.03)	0.652
Bladder cancer	116	0.3%	117	0.3%	233	0.948	1.03(0.80–1.33)	0.830
Renal cancer	98	0.3%	128	0.4%	226	0.053	1.33(1.02–1.73)	0.034
Thyroid cancer	71	0.2%	63	0.2%	134	0.545	0.89(0.64–1.25)	0.514
Skin cancer	137	0.4%	137	0.4%	274	1.000	1.02(0.81–1.30)	0.842
All cancers	2924	8.3%	3052	8.7%	5976	0.086	1.07(1.02–1.12)	0.010

* According to the data protection policy of NHIRD, the data on cancers with <3 cases cannot be provided.

## Data Availability

The NHIRD protects personal electronic data by rigorous confidentiality guidelines. The results presented in the study are available from the NHIRD of Taiwan for researchers who meet the criteria for access to confidential data, which cannot be shared publicly because of legal restrictions imposed by the government of Taiwan under the “Personal Information Protection Act”. Requests for data can be sent as a formal proposal to the NHIRD (https://dep.mohw.gov.tw/dos/np-2497-113.html, accessed on 1 August 2022). The contact information for needed data is: 886-2-85906828; Email: sthuiying@mohw.gov.tw.
